# Variation in brown rat cranial shape shows directional selection over 120 years in New York City

**DOI:** 10.1002/ece3.6228

**Published:** 2020-04-15

**Authors:** Emily E. Puckett, Emma Sherratt, Matthew Combs, Elizabeth J. Carlen, William Harcourt‐Smith, Jason Munshi‐South

**Affiliations:** ^1^ Department of Biological Sciences University of Memphis Memphis TN USA; ^2^ Department of Biological Sciences Louis Calder Center‐Biological Field Station Fordham University Armonk NY USA; ^3^ Department of Ecology and Evolutionary Biology School of Biological Sciences The University of Adelaide Adelaide SA Australia; ^4^ Division of Paleontology American Museum of Natural History New York NY USA; ^5^ Department of Anthropology The Graduate Center City University of New York New York NY USA; ^6^ Lehman College City University of New York Bronx NY USA; ^7^ Present address: Department of Ecology, Evolution and Environmental Biology Columbia University New York NY USA

**Keywords:** geometric morphometrics, rodent, urban evolution

## Abstract

Urbanization exposes species to novel environments and selection pressures that may change morphological traits within a population. We investigated how the shape and size of crania and mandibles changed over time within a population of brown rats (*Rattus norvegicus*) living in Manhattan, New York, USA, a highly urbanized environment. We measured 3D landmarks on the cranium and mandible of 62 adult individuals sampled in the 1890s and 2010s. Static allometry explained approximately 22% of shape variation in crania and mandible datasets, while time accounted for approximately 14% of variation. We did not observe significant changes in skull size through time or between the sexes. Estimating the P‐matrix revealed that directional selection explained temporal change of the crania but not the mandible. Specifically, rats from the 2010s had longer noses and shorter upper molar tooth rows, traits identified as adaptive to colder environments and higher quality or softer diets, respectively. Our results highlight the continual evolution to selection pressures. We acknowledge that urban selection pressures impacting cranial shape likely began in Europe prior to the introduction of rats to Manhattan. Yet, our study period spanned changes in intensity of artificial lighting, human population density, and human diet, thereby altering various aspects of rat ecology and hence pressures on the skull.

## Graphical abstract

Urbanization exerts unique selective pressures on wildlife populations. We observed directional changes in brown rat skull shape over 120 years for the population living in Manhattan, New York.

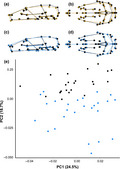

## 1 INTRODUCTION

Urbanization has dramatically changed natural landscapes and exposed wildlife to unique selection pressures including but not limited to increased impervious surface area, increased temperatures due to heat island effects, aerial and subterranean infrastructure, altered light–dark cycles via artificial lighting, increased noise, altered diets composed of highly processed human foods, exposure to toxins, introduction to novel predators and/or release from predation, and increased contact rates with humans. These selection pressures have changed the morphology, behavior, patterns of gene flow, mutation rates, and allele frequencies at both neutral and selective sites across diverse species inhabiting urban landscapes (Alberti, 2015; Johnson & Munshi‐South, 2017).

Head morphology (particularly the underlying skull shape and size) impacts how animals interact with their environment through, for example, visual acuity, olfaction, nasal heat dissipation, brain size, and diet. Evolvability (i.e., the ability of a trait to track a selective gradient; Linde‐Medina, Boughner, Santana, & Diogo, 2016; Marroig, Shirai, Porto, de Oliveira, & De Conto, 2009) of head morphology has been attributed to genetic drift, natural selection, phenotypic plasticity, or a combination of these factors depending on the taxa and trait of interest (examples below). Studies of evolvability generally take two approaches, either examining closely related species or using time‐series sampling to track a population.

Genetic drift in genes coding for morphological variation can result in phenotypic changes; thus, drift serves as a null hypothesis for morphological change between taxa (Marroig & Cheverud, 2004). This drift in morphology explained most of the global variation in human cranial shape and size when isolation‐by‐distance was taken into account (Betti, Balloux, Hanihara, & Manica, 2010). Drift also explained ventral cranial differences across the range of Martino's vole (*Dinaromys bogdanovi*; Kryštufek, Klenovšek, Bužan, Loy, & Janžekovič, 2012). While many studies test for drift, there are few examples that failed to reject the null model; thus, it is unclear to what extent these results may be publication bias or a function of the true prevalence of this evolutionary process within the systems investigated.

Natural and artificial selections both influence cranial shape. Low temperatures were associated with long and narrow noses, and wider crania in humans (Betti et al., 2010; Zaidi et al., 2017). Additionally, smaller sinus volumes were found in human and macaque populations in colder environments (Rae, Hill, Hamada, & Koppe, 2003; Shea, 1977). Assis, Patton, Hubbe, and Marroig (2016) demonstrated cranial morphology changes in two chipmunk species, one shifting altitudinally over time (*Tamias alpinus*), and one that did not shift its range (*T. speciosus*) and was susceptible to increasing temperatures. Using temporal samples covering approximately 95 years, the authors observed the distance between nasale and the intradentale superior, and nasale and nasion increased over time, suggesting heat dissipation as a possible mechanism for the morphological change (Assis et al., 2016). Shifts in diet also affect cranial and mandible morphology. Urban populations of white‐footed mice (*Peromyscus leucopus*) had shorter tooth rows than their rural counterparts (Yu, Munshi‐South, & Sargis, 2017), and urban populations of house finches (*Carpodacus mexicanus*) had larger bills with higher bite force than a nearby rural population (Badyaev, Young, Oh, & Addison, 2008). Food hardness explains both of these results as urban mice have access to softer foods thus requiring less chewing surface, and urban finches have access to harder seeds (e.g., sunflower seeds in bird feeders) requiring more bite force and thus a shift in beak shape. A temporal study of bats (*Pipistrellus kuhlii*) observed an increase in cranial size without an increase in postcranial body size and suggested that a shift to foraging on moths gathered near artificial light was a driver for this change (Tomassini, Colangelo, Agnelli, Jones, & Russo, 2014). Finally, a temporal study of cranial shape in the St. Bernard breed of domestic dogs (*Canis familiaris*) identified that skull shape shifted over time to align with the written ideal breed traits, providing an example of artificial selection on crania (Drake & Klingenberg, 2008), which may also occur in other breeds albeit undocumented. Thus, multiple systems experienced rapid morphological change due to anthropogenic selective forces.

Phenotypic plasticity has been proposed as an alternative explanation to selection for morphological changes in urban environments (Snell‐Rood & Wick, 2013), and both laboratory and field studies observed plastic responses to selective agents of interest. Brown rats (*Rattus norvegicus*) reared in cold temperatures (5°C) had smaller sinus and nasal cavity volumes than individuals reared at room temperature (22°C), where the latter trait was in the opposite direction of primate results, yet the authors noted that phenotypic plasticity in the lab may have produced results that differed from natural populations under selection (Rae, Viðarsdóttir, Jeffery, & Steegmann, 2006). This experiment also observed a reduction in cranial but not postcranial body size in the cold‐reared rats (Rae et al., 2006). Food hardness also resulted in plastic variation as laboratory mice fed a soft diet of ground pellets in jelly had shorter coronoid and angular processes, and posteriorly shifted incisors and molars when compared to mice fed whole pellets (Anderson, Renaud, & Rayfield, 2014). The change from a hard to soft diet released mouse mandibles from developmental constraints and decreased the mechanical load (Anderson et al., 2014). Environmental complexity has also been associated with cranial plasticity. Zebrafish (*Danio rerio*) housed in tanks with plastic plants, shelter, gravel, and a novel object had larger brains than those in tanks without enrichment (DePasquale, Neuberger, Hirrlinger, & Braithwaite, 2016). Similarly, populations of white‐footed mice and meadow voles (*Microtus pennsylvanicus*) had increased cranial capacity in urban compared with rural sites (Snell‐Rood & Wick, 2013). The same study also observed increased cranial capacity over time in rural populations of big brown bats (*Eptesicus fuscus*), little brown bats (*Myostis lucifugus*), masked shrews (*Sorex cinereus*), and northern short‐tailed shrews (*Blarina brevicauda*; Snell‐Rood & Wick, 2013). The authors hypothesized that increased environmental complexity (whether due to the contrast between urban [i.e., complex] and rural [i.e., simple] landscapes, or an increase in complexity within rural environments over time) was the selection pressure for increased cranial capacity which increased fitness. Increased cranial capacity in birds has been linked to greater cognitive ability and colonization of novel environments (Sol, Duncan, Blackburn, Cassey, & Lefebvre, 2005).

We investigated whether brown rat skulls changed in either shape or size over time in the borough of Manhattan, New York City, New York, USA. Brown rats were introduced to Manhattan around 1,750 when urbanization was contained to the southern portion of the island. The entire island is now highly urbanized containing above and below ground built environments, with pockets of green space. It is home to 1.58 M people that can increase to 3.94 M with daily commuters and tourists (Moss & Qing, 2012); human population density has been associated with increases in rodent total body length, hind foot length, and distance from superorbital to nasale (Pergams & Lawler, 2009). As a human commensal, brown rats receive most of their food, water, and shelter by living within cities or farms where many resources are human‐derived. The evolution of commensalism may have released brown rats from their natal range in northern China and Mongolia, where the animals had to be cold hardy, and facilitated their global invasion across multiple climatic zones (Puckett & Munshi‐South, 2019). We used geometric morphometrics to compare cranial and mandible shape and size of rats collected between 1889–1895 and 2014–2016. Under an assumption that rats breed three times annually (based upon length of pregnancy and weaning), our study investigated evolutionary change over 355–380 generations. Population genomic and demographic analyses identified rats in Manhattan as a single genetic population that has had limited gene flow since establishment (Combs, Puckett, Richardson, Mims, & Munshi‐South, 2018; Puckett et al., 2016). We had three predictions regarding the direction of evolutionary morphological change due to pressures from intensifying urbanization: (a) braincase size would increase over time for increased cognition in a complex landscape; (b) the nose would shorten over time to increase heat exchange given urban heat island effects; and (c) tooth row length would decrease over time as rats ate increased amounts of softer foods. We observed changes in cranial and mandible shape over time and then tested whether this was due to drift or selection.

## 2 MATERIALS AND METHODS

### 2.1 Data collection

Samples for this study came from two different sources, an existing collection of rats captured across NYC and housed at the American Museum of Natural History, New York, USA (1890s samples), and a new collection of specimens housed at the Yale Peabody Museum (2010s samples). The latter were part of an investigation into the population genomics of brown rats in Manhattan, USA, where 393 individuals were captured and euthanized (Fordham University IACUC JMS‐13‐02; Combs et al., 2018); of these, 44 rats were prepared as museum specimens. From these two collections, we only included adults defined by the full eruption of the third molar and individuals with known sex (Table S1). We recorded the sex and year of collection and then grouped years into a categorical variable “time period” with two levels (1890s for samples collected from 1889–1895; and 2010s for samples collected from 2014–2016; Table S1). We used 3D geometric morphometrics to characterize the shape of the crania and mandibles. A single author (EEP) collected the morphometric data with a Microscribe 3D Digitizer (Solution Technologies Inc.) on 43 and 15 homologous landmarks, respectively, on the crania and mandibles (Figures S1 and S2, Tables S2 and S3). For the crania, 18 and 30 landmarks were digitized, respectively, in the dorsal and ventral orientations with five common landmarks between them, then combined into a single configuration using MORPHEUS (Slice, 2013). We imputed missing landmarks using a thin‐plate spline approach with the function “estimate.missing” in the R package *geomorph* v.3.0.5 (Adams, Collyer, Kaliontzopoulou, & Sherratt, 2017). The landmark coordinates for crania and mandibles were aligned separately using a generalized Procrustes superimposition, where we used “bilat.symmetry” on the crania to account for the object symmetry of the skull, but “gpagen” for the mandible as only the left side was measured (Klingenberg, Barluenga, & Meyer, 2002). The resulting Procrustes shape variables for both the crania and mandibles were used for all subsequent analyses and performed using *geomorph* unless otherwise stated. We calculated cranium and mandible size independently from the landmark configurations using the centroid size (the square root of the sum of the square distances of each landmark from the centroid).

### 2.2 Error estimation

Two specimens were selected, one each from the 1890s and 2010s time periods, and measured 10 times, interspersed between specimen measurements, and then used to estimate measurement error. We used a Procrustes ANOVA to calculate the percentage of error from digitizing using the “procD.lm” function. Digitizing error was minimal: the dorsal, ventral, and mandible error values were, respectively, 1.8%, 1.9%, and 4.2%.

### 2.3 Morphometric analyses

We tested for changes in cranial or mandible shape between the two time periods, while controlling for variation due to sex (sexual shape dimorphism) and size (static allometry), by performing a Procrustes analysis of covariance (ANCOVA) using the “advanced.procD.lm” function in *geomorph* with type I error and the explanatory variables log‐transformed centroid size, time period, and sex. Statistical significance was evaluated using the *F*‐ratio test (Goodall, 1991) with a randomized residual permutation procedure (RRPP; Collyer, Sekora, & Adams, 2014) and 1,000 iterations. To quantify the amount of shape variation explained by the interaction of time period and sex, we ran the “advanced.procD.lm” function to compare the full model (log centroid size × time period × sex) to a reduced model only accounting for size. Similarly, we tested for a change in size of the crania and mandibles through time using a Procrustes ANCOVA with only time period, sex, and an interaction term as explanatory variables. For both the crania and mandible datasets, we visualized the shape variation among all specimens using principal components (PC) analysis.

### 2.4 Evolutionary mode

We investigated whether morphological shape change in both datasets was due to genetic drift or directional selection between the time periods by comparing phenotypic (**P**) variance–covariance matrices. For mammalian skull evolution, the **P**‐matrix has repeatedly been shown to be a proxy for the genotypic (**G**) matrix (Cheverud, 1988; Marroig & Cheverud, 2004; Roff, 2000), although it may be a poor model when trait heritability is low. We made the **P**‐matrices by calculating interlandmark distances (Tables S2 and S3). For pairs of landmarks with bilateral symmetry on the cranium, we calculated the average distance between the two sides. We compared the variance–covariance structure of the 1890s and 2010s matrices using the random skewers method (Marroig & Cheverud, 2001) with 1,000 random vector draws, implemented in the R package *EvolQG* v.0.2.5 (Melo, Garcia, Hubbe, Assis, & Marroig, 2016).

We ran 1,000 iterations of the multivariate drift test in *EvolQG* to simulate the amount of morphological divergence expected under a model of genetic drift. Specifically, this test creates a divergence matrix

$$D=G(t/N_{e})$$

where *G* is the historic **G**‐matrix, which we will substitute with the historic **P**‐matrix; *t* is the number of generations between the time points; and *N*_e_ is the effective population size (Assis et al., 2016). The “MultivDriftTest” function in *EvolQG* then computes a multivariate normal distribution and compares the expected values between the time points to the observed value and calculates a confidence interval expected under a scenario with drift. Both of our sampling periods covered multiple years, and we used the estimate of three rat generations per year (Davis, 1953). Thus, the data spanned 357–381 generations, and we selected the mean 370 generations as the value for *t*. To estimate *N*_e_, we first generated a folded site frequency spectrum (SFS) in ANGSD v0.915 (Korneliussen, Albrechtsen, & Nielsen, 2014) from a dataset of rats collected in NYC from 2014 to 2016 and genotyped using double digest restriction associated sequencing (ddRAD‐Seq; Combs et al., 2018). Due to computational limits, we selected 248 individuals at random from the full dataset to estimate the SFS. We built a demographic model of a single population allowing for population growth or contraction in fastsimcoal2 v2.5.2.21 (Excoffier & Foll, 2011) with a minimum 1 × 10^5^ and maximum 2 × 10^5^ simulations per iteration. We estimated *N*_e_ (prior 10^0^–10^4^ individuals) and growth rate (*r*) where each parameter was estimated following a minimum of 10 and maximum of 50 ECM cycles and was terminated when the maximum composite likelihood between two iterations was <0.001. (See Appendix S1 for fastsimcoal2 input files.) From the 50 iterations, we selected the model with the highest estimated likelihood and used the estimated *N_e_* from that model, understanding that this point estimate was a single iteration of the coalescent process. We ran 1,000 iterations of the multivariate drift test to simulate an expected distribution of drift values to compare to the observed value of our **D**‐matrix.

## 3 RESULTS

### 3.1 Cranial shape variation

We observed significant differences in cranial shape for the main effects related to centroid size, time period, and sex that accounted for 21.2%, 13.8%, and 1.4% of the respective variation in the data (Table 1). We tested for differences (homogeneity of slopes) between pairwise combinations of the time period and sex factors and observed no significant differences for linear model slope vector lengths, meaning there were no groups that had increased allometric change as they grew larger. Similarly, no groups differed in their slope angles, thus rats’ cranial shape changed in the same way as they grew larger (Table S4).

**TABLE 1:** Results of the Procrustes ANCOVA for cranium and mandible shape variation accounting for centroid size (Size) and assessing the impact of Time Period (1890s and 2010s), Sex (female and male), and the two‐way and three‐way interaction terms

	*df*	SS	MS	*R* ^2^	*F*	*Z*	*p*
Cranium							
Log(Size)	1	0.016	0.016	.213	14.868	6.682	**.001**
Time Period	1	0.011	0.011	.141	9.838	7.253	**.001**
Sex	1	0.001	0.001	.014	1.014	1.726	**.049**
Log(Size) × Time Period	1	0.001	0.001	.012	0.829	1.076	.133
Log(Size) × Sex	1	0.001	0.001	.012	0.836	1.109	.128
Time Period × Sex	1	0.001	0.001	.010	0.730	0.700	.249
Log(Size) × Time Period × Sex	1	0.001	0.001	.012	0.826	1.177	.119
Residuals	41	0.044	0.001	.586			
Total	48	0.075					
Mandible							
Log(Size)	1	0.049	0.049	.228	19.577	7.276	**.001**
Time Period	1	0.030	0.030	.138	11.852	7.761	**.001**
Sex	1	0.004	0.004	.019	1.615	3.165	**.001**
Log(Size) × Time Period	1	0.003	0.003	.014	1.227	2.325	**.008**
Log(Size) × Sex	1	0.002	0.002	.011	0.905	1.430	.072
TimePeriod × Sex	1	0.001	0.001	.006	0.539	−0.077	.543
Log(Size) × Time Period × Sex	1	0.003	0.003	.015	1.285	2.627	**.002**
Residuals	49	0.123	0.003				
Total	56	0.215					

Note

Variables in bold were significant with an *α* of 0.05. Pairwise group comparisons are provided in Table S4.

**FIGURE 1:**
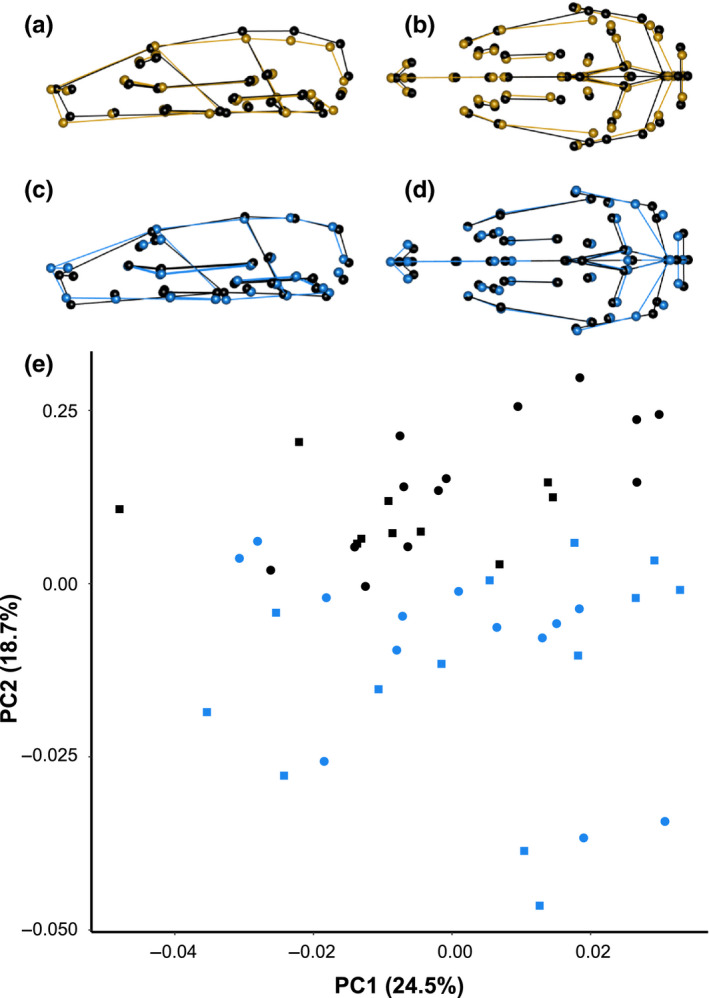
Changes in brown rat morphometric shape along principal component (PC) axes representing PC1 (a, b) and PC2 (c, d) for lateral (a, c) and overhead (b, d) views. Wireframe models show the shape differences between PC score minima (color, gold or blue) and maxima (black). (e) PCA of cranial shape of brown rats from Manhattan, USA. Color represents time periods (black—1890s; blue—2010s) and shape represents sex (circles—females; squares—males). PC1 represents the static allometric axis, with larger animals on the right

A scatterplot of the first two PC axes clearly show how shape variation was structured among our factors of interest (Figure 1e). The first PC axis explained 24.5% of the variance and described shape changes associated with size. Rats with high PC1 scores represented large animals and had a shorter snout, a more rounded cranial midline, posteriorly extended lambda and opisthion, wider zygomatic arches, a wider and taller foramen magnum, and longer molar tooth rows (Figure 1a,b). The second PC axis explained 18.7% of the variance and described shape changes associated with time (Figure 1e), separating the 1890s and 2010s groups with almost no overlap. High PC2 scores were associated with the samples from the 1890s where crania had a sharper angle between nasale and nasion, a more posteriorly shifted nasale and intradentale superior, a posteriorly shifted foramen magnum, an anterior external auditory meatus shifted toward the midline, anteriorly shifted anterior superior alveoli, and a longer molar tooth row (Figure 1c,d). There was no visible separation between the time periods on any other PC axes.

### 3.2 Mandible shape variation

We observed significant differences in mandible shape for the three‐way interaction between log size, time period, and sex, as well as the two‐way interaction for log size and time period (Table 1). The significant three‐way interaction indicated that there were allometric differences between sexes between time periods; similarly, the significant two‐way interaction in the mandibles indicated allometric differences between time periods without regard to sex. The three main effects were also significant and accounted for 22.7%, 13.8%, and 1.9% of the variance in the data for log‐transformed size, time period, and sex, respectively. The homogeneity of slopes test showed no significant difference for any pairwise groups of time period and sex in either slope vector length or angles between slope vectors (Table S4).

**FIGURE 2:**
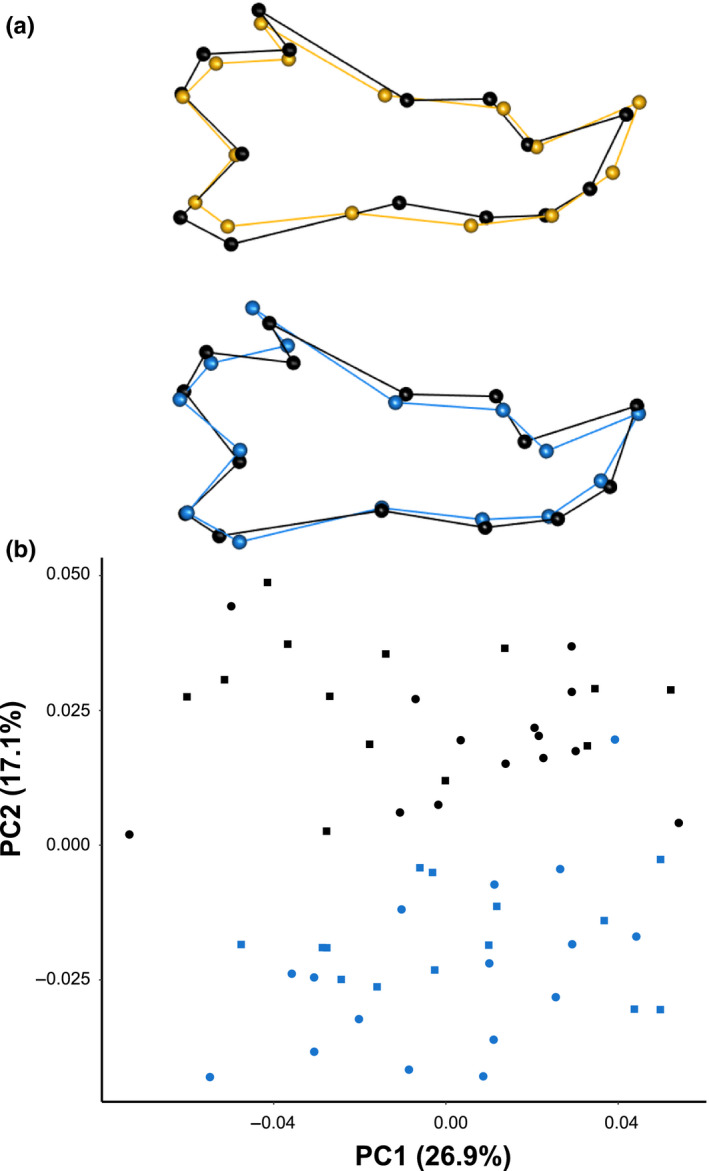
Changes in brown rat mandible shape along principal component (PC) axes representing (a) PC1 and (b) PC2. Wireframe models show the shape differences between PC score minima (color, gold or blue) and maxima (black). (c) PCA of mandible shape of brown rats from Manhattan, USA. Color represents time periods (black—1890s; blue—2010s) and shape represents sex (circles—females; squares—males). PC1 represents the static allometric axis, with larger animals on the right

The first PC axis described 26.9% of the variance and, like the cranial dataset, also described shape changes associated with static allometry (Figure 2c). High PC1 scores represented larger animals and reflected a shorter corpus, a wider angle of the condylar process, greater length of the angular process, and a more curved line of the angular process and corpus (Figure 2a). The second PC axis described 17.1% of the variance and separated rats into two groups corresponding to time period. High PC2 scores represented the 1890s samples and were distinguished by an increase in the curvature of the posterior corpus, a shallower angular process, a shorter molar alveolus, and a shorter coronoid process (Figure 2b). Similar to the crania, there was no visible separation between the time periods on any other PC axes.

### 3.3 Size variation

We investigated how time period and sex influenced log centroid size and observed no significant differences for main effects or the interaction term (Table 2). Thus, size in rat crania and mandibles was not significantly different through time, nor due to sexual dimorphism.

**TABLE 2:** Results of the Procrustes ANCOVA for variation in cranium and mandible size (centroid size) assessing the impact of Time Period (1890s and 2010s), Sex (female and male), and the two‐way interaction term

	*df*	SS	MS	*R* ^2^	*F*	*Z*	*p*
Cranium							
Time Period	1	11.370	11.375	.004	0.181	−0.216	.660
Sex	1	4.420	4.421	.001	0.070	−0.565	.777
Time Period × Sex	1	126.610	126.610	.043	2.014	0.853	.172
Residuals	45	2,829.240	62.872	.952			
Total	48	2,971.650					
Mandible							
Time Period	1	15.120	15.117	.019	1.039	0.579	.311
Sex	1	2.520	2.517	.003	0.173	−0.183	.647
Time Period × Sex	1	15.930	15.926	.020	1.094	0.575	.317
Residuals	53	771.380	14.554				
Total	56	804.930					

Note

No variables were significant with an *α* of 0.05.

### 3.4 Evolutionary mode

We estimated a contemporary *N*_e_ of 24,905 individuals and *r* of 6.85 × 10^–11^, which indicated very little population size change over time. This varies from previous work on *N_e_* in Manhattan that estimated 259 individuals (Combs et al., 2018), yet that estimate was likely biased given the diffuse geographic sampling approach and use of NeLD (Do et al., 2014; Gilbert & Whitlock, 2015). Our estimate of *N*_e_ 24,905 is reasonable given an estimated census size of 250,000 individuals across NYC; we acknowledge that estimate includes all five boroughs (Davis, 1950) while our work is confined to Manhattan, although the presence of population structure is likely within the same evolutionary lineage (Puckett et al., 2016).

Using the random skewers method, we observed matrix correlations between the historic and contemporary datasets of 0.73 and 0.92 for the cranium and mandible datasets, respectively, meaning that the two temporal datasets would respond similarly to an applied selection pressure. Both correlations were significant (*P*_cranium_ = .001, *P*_mandible_ < .001); thus, the matrices were more similar than expected by chance. We rejected the null hypothesis of shape change due to genetic drift over time for the cranium, as the observed **D**‐matrix (1.43–3.06) was greater than the confidence intervals of the simulated distribution (0.14–1.16), suggesting directional selection. However, for the mandible, we failed to reject the null hypothesis as the **D**‐matrix (1.00–2.46) overlapped with the simulated distribution (0.13–1.32) suggesting drift.

## 4 DISCUSSION

We observed that brown rat crania and mandibles have significantly changed in shape, but not size, over the last 120 years in Manhattan, USA (Figures 1 and 2). Our evolutionary mode analysis suggests that shape changes were due to directional selection in the cranium and drift in the mandible. In the crania, we observed a slightly smaller hindbrain case, longer snout, shorter upper molar tooth row, and a shift in the ear canal in the contemporary compared to historic samples (Figure 1c,d), which we discuss in detail below.

### 4.1 Cranial morphological change

#### 4.1.1 Braincase

We hypothesized that braincase volume would increase over time as rats adapted to a complex urban environment. Although the braincase evolves slowly across mammalian species it has high evolvability (i.e., the capacity to evolve in the direction of the selection gradient; Linde‐Medina et al., 2016). Brain size has been positively correlated with both the number of species present (Pollen et al., 2007) and the structural complexity of the abiotic environment (DePasquale et al., 2016). We observed that rats with larger crania (high cranial PC1 scores) had more curved and wider hind‐skulls (Figure 1) suggestive of a larger volume; however, PC1 was associated with allometric variation and did not display temporal variation. Along PC2, we observed that the brain case was shallower and the foramen magnum narrower in the 2010s population (Figure 1). Therefore, our results suggest that brain size did not increase over time.

Snell‐Rood and Wick (2013) proposed that braincase size may plastically increase as an initial response to invading an urban environment, then decrease following acclimatization. Our results neither support nor refute this hypothesis as we only had access to skulls from two time points, so were unable to test for a parabolic trend. Snell‐Rood and Wick's study (2013) occurred in a less urbanized area than Manhattan; therefore, they may have identified plasticity in the dataset due to increasing urbanization. Populations within city centers may live in a stable environment, as evidenced by the limited distance rats (Byers, Lee, Patrick, & Himsworth, 2019) and other species (Tucker et al., 2018) travel in urban habitats; thus, their lives entail less risk, problem‐solving, and time spent searching for food than populations in unstable or disturbance prone habitats. Suburban and exurban populations experience greater temporal change as urbanization occurs then intensifies.

#### 4.1.2 Nasal cavity

Both inter‐ and intraspecies differences in nasal cavity volume have been observed in mammals. Chipmunk populations in a warming environment showed a wider opening of the nasal cavity, similarly an experimental population of rats reared at room temperature had larger maxillary sinus and nasal cavity volumes than those reared in a colder environment (Assis et al., 2016; Rae et al., 2006). Larger volumes can be achieved through the elongation of nasale to nasion and/or nasale to intradentale superior. We observed a longer nasale to nasion in the 2010s sample (Figure 1d), suggestive of an expanded nasal cavity and thus the opposite of our prediction of a shorter nose due to increasing temperatures over time from urban heat island effects. However, rats are a fossorial species that spend the first six weeks of life in underground soil burrows or sewer infrastructure which may buffer heat island effects. We also observed natural variation in this trait along the allometric axis PC1 (Figure 1e). Alternatively, this variation could represent plasticity related to the ambient temperature following birth, consistent with experimental results (Rae et al., 2006).

#### 4.1.3 Tooth row

We observed that the upper tooth row was larger for rats with higher PC1 scores and longer for rats with higher PC2 scores (Figure 1), where the latter was associated with the 1890s samples. Longer tooth rows are a proxy for increased chewing surface area, which is associated with low food quality (i.e., bark) and/or harder foods. We observed that tooth row length decreased over time within this urban brown rat population (Figure 1d). We measured tooth row length and observed that the mean difference between time points was 0.19 mm. A rural‐urban gradient study of *Peromyscus leucopus* cranial morphology observed similar results, where the urban population had shorter tooth rows than the rural population (Yu et al., 2017). A study of collagen isotopes in brown rats in and around Toronto, Ontario, sampled from 1,790 to 1,890 found that the urban population had higher ^15^N to ^14^N ratios compared to the rural population, which is indicative of higher quality diets with increased amounts of animal proteins (Guiry & Buckley, 2018). Our data indicate a lengthening of the rostrum in the 2010s sample (Figure 1c,d) which has been associated with shifts to carnivorous or insectivorous diets in rodents (Renaud et al., 2018; Samuels, 2009). The tooth row data suggest that urban rats and mice ate higher quality and/or softer foods; however, these explanations are not mutually exclusive, as anthropogenic foods can be both calorically dense and soft.

### 4.2 Mandible morphological change

Studies of the plasticity of mouse mandibles fed diets of varying hardness observed that animals on the hard diet had extended coronoid and angular processes, longer molar row, and ventrally shifted molar alveolar (Anderson et al., 2014; Renaud, Auffray, & de la Porte, 2010). We observed a dorsally extended coronoid process and slightly lower incisor alveolar region in the 2010s samples when compared to the 1890s; however, we did not measure molar row. If our results regarding shape changes were related to rat diets, then that suggests contemporary rats ate harder foods than in the past. This finding contrasts with our interpretation of the shortening of upper tooth row length in contemporary urban diets being softer due to processed and cooked human foods. Notably, we would not expect results as strong as in the experimental study as urban rat diets likely consist of a variety of anthropogenic and natural foods that vary in hardness.

### 4.3 Temporal change in morphology within urban environments

Our data suggest that temporal morphological change of brown rat cranial shape in this population was driven by selection to the urban environment. Previous work on brown rat cranial morphometrics identified moderate correspondence between the **P**‐ and **G**‐matrixes such that 25%–68% of measured features were due to additive genetic variance (Atchley, Rutledge, & Cowley, 1981). Thus, it is possible that some of the variation in our study was the result of phenotypic plasticity. We did not collect data to identify selection pressures from the urban environment for these morphometric changes; however, experimental results suggest dietary changes for tooth row length and adjusting to environmental complexity for brain case size could be factors influencing temporal shape change (see Section 4). Our hypothesis that selection pressures within the urban environment would result in directional change in cranial shape was partly based upon an assumption that urbanization pressure intensified from the 1890s to 2010s. This assumption deserves two critiques. First, rats were introduced to NYC around 1,750 from port cities in western Europe (Armitage, 1993); thus, some morphological change related to urbanization may have occurred in Europe, in which case the historic specimens may have already changed in shape due to urbanization. Second, our study site was already highly urbanized at the time when the historic samples were collected (late 1890s). The gridded road network in Manhattan was designed in 1811 and installed over the next 60 years. The first electric streetlights were installed in 1880 ultimately leading Broadway to receive its nickname, “The Great White Way,” due to the brightness of the lights in the 1890s. Finally, census records since 1900 show the height of Manhattan's population at 2.33 M people in 1910 following massive immigration from Europe, that since declined to 1.58 M in 2010, although daily commuters and tourists increase the number of people on the island and thus the effective density of humans that wildlife encounter. Thus, we acknowledge that substantial morphological change occurred prior to the beginning of our study. In addition to shifts in the built environment, human behavior patterns could be included as variable factors influencing rats’ environment throughout the 20th century. Specifically, human diets shifted to have increasing sugars and fats (Cordain et al., 2005), along with increasing proportions of processed foods may have changed the caloric composition of refuse for which rats had access. Evans, Campbell, and Murcott (2012) note a shift in the 1950s from food scarcity to abundance that may have changed the amount of waste produced, but in an unknowable way given scholarship and data on food waste practices over time. Concurrent to shifts in diet were changes in sanitation; specifically, effective sanitation in Manhattan began in 1895, before which refuse was primarily dumped in the river and streets were piled with general dirt, animal excrement, dead animals, food waste, and wood products (Nagel, 2013). Thus, these factors may have influenced the contemporary urban environment over which we observed rapid evolution in rats.

Studies of morphological change over time due to urbanization have observed variation in the temporal patterns of cranial shape change. A study of white‐footed mice cranial shape in Chicago, USA, between 1903 and 2003 identified change in shape between 1976 and 2001 due to population replacement (Pergams & Lacy, 2008); similar to our work, the urban environment may not have changed much during this time, opening the question of what landscape processes facilitated replacement. A study of temporal change in fish body shape within urbanizing streams observed rapid change followed by maintenance of the new shape over time (Kern & Langerhans, 2018). In contrast, Snell‐Rood and Wick (2013) hypothesized a parabolic plastic response in cranial morphology, particularly brain case size, when initially encountering urbanization that may decrease as a population adjusts to the urban environment. Unfortunately, rat samples from NYC do not extend further back in time and there are few temporal samples between the 1890s and 2010s collections (and none with large sample sizes) for us to test whether shape change followed a logistic or parabolic curve. It may be likely that rat cranial shape evolved advantageous morphologies rapidly upon establishing a commensal relationship with humans and then were maintained. Yet we note that shape changes of individual structures may vary through time, and see this as particularly relevant to our results on tooth row length and contemporary shifts in diets of urban rats. Given spatial, temporal, and sample size limitations of historic collections, the best approach to quantify morphological change may be to sample species that live in areas where new cities are currently being built. Sampling species and selection pressures now and in the future will allow for explicit tests of morphological change in response to habitat change, thus enabling inference on how urbanization affects diverse species’ abilities to interact with their environments.

## CONFLICT OF INTEREST

The authors have no conflicts of interest.

## AUTHOR CONTRIBUTION

**Emily E. Puckett:** Conceptualization (lead); Formal analysis (equal); Investigation (lead); Methodology (supporting); Project administration (lead); Visualization (lead); Writing‐original draft (lead); Writing‐review & editing (equal). **Emma Sherratt:** Formal analysis (supporting); Methodology (equal); Writing‐review & editing (equal). **Matthew Combs:** Resources (equal); Writing‐review & editing (equal). **Elizabeth J Carlen:** Resources (equal); Writing‐review & editing (equal). **William Harcourt‐Smith:** Formal analysis (supporting); Methodology (equal); Resources (supporting); Writing‐review & editing (equal). **Jason Munshi‐South:** Funding acquisition (lead); Resources (equal); Writing‐review & editing (equal). 

## Supporting information

Appendix S1

## Data Availability

Microscribe data for crania and mandibles, and the SFS of contemporary NYC rats available on Data Dryad https://doi.org/10.5061/dryad.g4f4qrfmn.
