# Supplementary material for: Variation in brown rat cranial shape shows directional selection over 120 years in New York City
Source: Ecol Evol. 2020 Apr 15;10(11):4739–48. doi: 10.1002/ece3.6228 (PMC7297766; doi:10.1002/ece3.6228)
Supplement: Supplementary file 1 — Appendix S1 [file ECE3-10-4739-s001.docx]

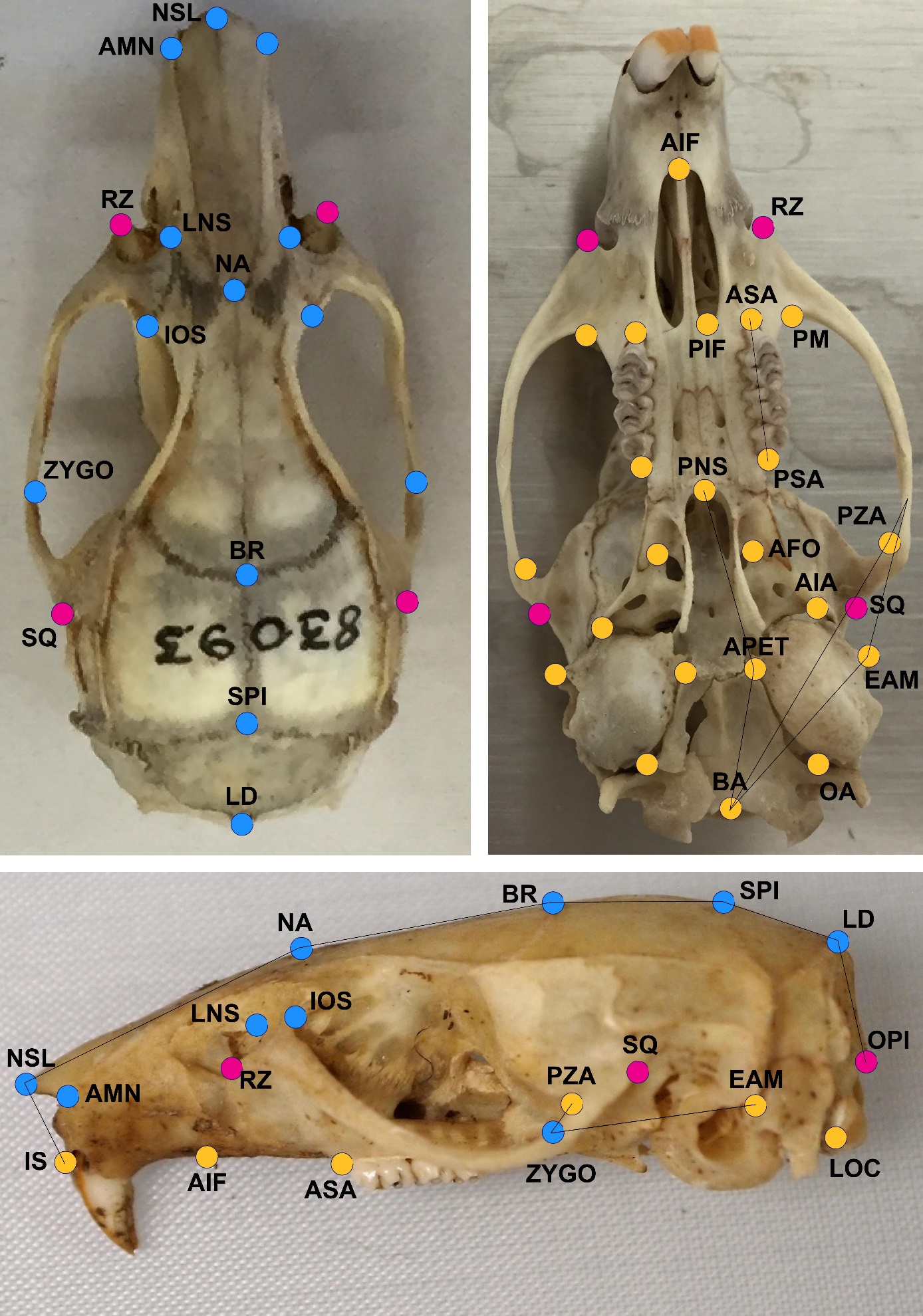
**APPENDIX**

**Figure S1-** Cranial landmarks (p=43) taken in the dorsal (A) and ventral (B) orientations with lateral view (C) shown for comparison. Blue, yellow, and pink dots identify landmarks taken in the dorsal, ventral, or both orientations, respectively. Interlandmark distances shown as thin lines between most landmarks (except width of LOC) for the left half of the cranium. See Table S2 for a list of abbreviations and landmark descriptions.


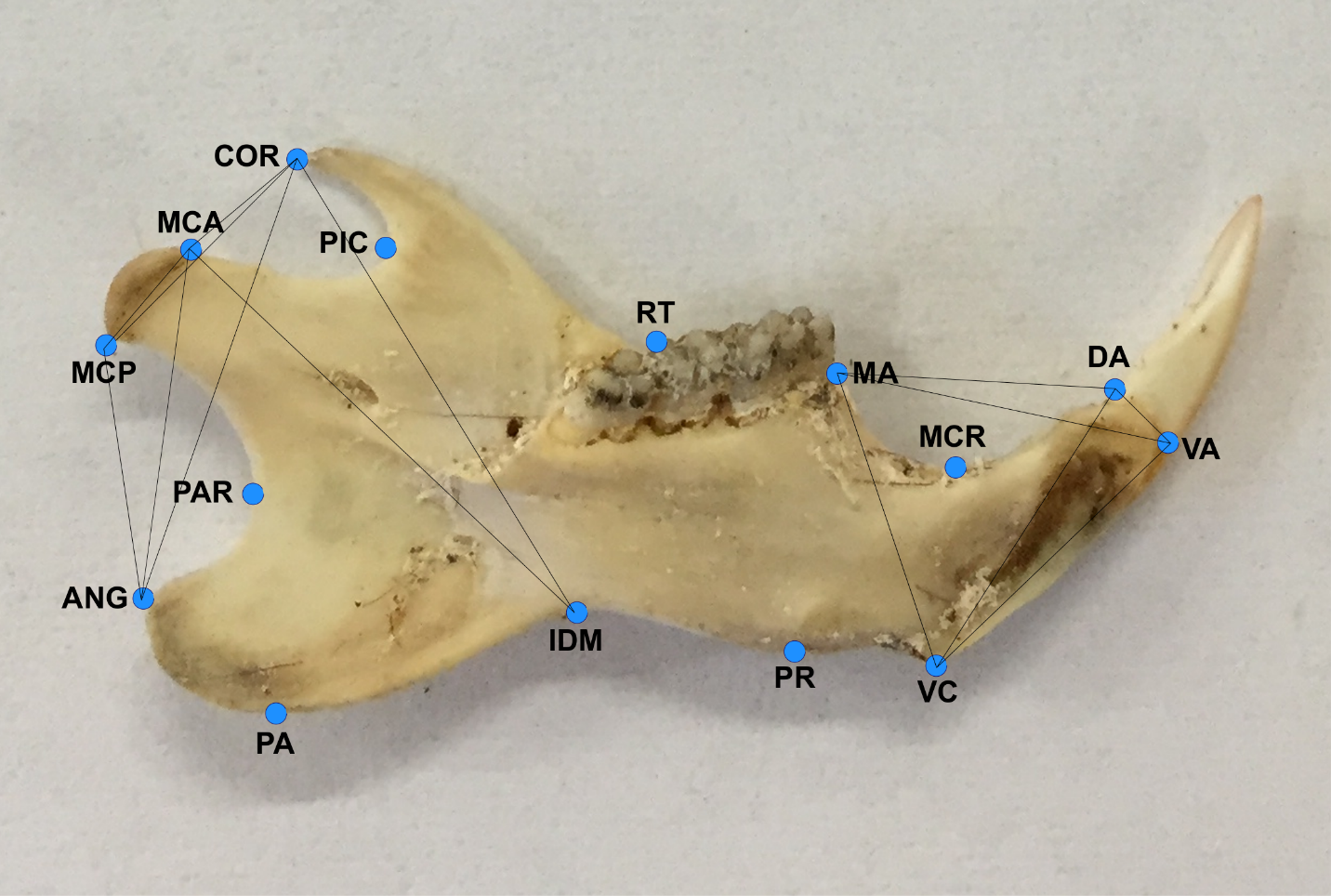


**Figure S2-** Mandible landmarks (p=15; blue dots) taken on the left mandible. See Table S3 for list of abbreviations, landmark descriptions, and interlandmark distances (thin black lines).

**Table S1**- Samples included in this study with accession numbers from either the American Museum of Natural History (AMNH) or Yale Peabody Museum (YPM), with the collection year, sex, and inclusion in the dorsal, ventral, and/or mandible datasets. For the cranium, we measured 49 specimens with 26 (14:12 F:M) in the 1890s group and 23 (9:14) in the 2010s group. For the mandible, we measured 57 specimens with 27 (14:13) in the 1890s and 30 (16:14) in the 2010s.

|  |  |  |  |  |
| --- | --- | --- | --- | --- |
| **Accession** | **Sex** | **Year** | **Cranium** | **Mandible** |
| AMNH1217 | M | 1889 | ✓ | ✓ |
| AMNH1219 | M | 1889 |  | ✓ |
| AMNH1220 | M | 1889 | ✓ | ✓ |
| AMNH1230 | M | 1889 |  | ✓ |
| AMNH1232 | F | 1889 |  | ✓ |
| AMNH1233 | M | 1889 | ✓ | ✓ |
| AMNH801 | F | 1889 | ✓ | ✓ |
| AMNH802 | F | 1889 | ✓ | ✓ |
| AMNH803 | F | 1889 | ✓ | ✓ |
| AMNH1605 | M | 1890 | ✓ | ✓ |
| AMNH1607 | M | 1890 | ✓ | ✓ |
| AMNH1609 | M | 1890 | ✓ | ✓ |
| AMNH1610 | M | 1890 | ✓ | ✓ |
| AMNH4603 | F | 1893 | ✓ | ✓ |
| AMNH5054 | M | 1893 | ✓ | ✓ |
| AMNH6132 | F | 1894 | ✓ | ✓ |
| AMNH6133 | M | 1894 | ✓ |  |
| AMNH6135 | M | 1894 | ✓ | ✓ |
| AMNH6136 | F | 1894 |  | ✓ |
| AMNH6138 | F | 1894 | ✓ | ✓ |
| AMNH6140 | F | 1894 | ✓ |  |
| AMNH6141 | M | 1894 | ✓ | ✓ |
| AMNH6142 | F | 1894 | ✓ | ✓ |
| AMNH6143 | F | 1894 | ✓ | ✓ |
| AMNH6144 | F | 1894 | ✓ | ✓ |
| AMNH6145 | F | 1894 | ✓ | ✓ |
| AMNH6147 | F | 1894 | ✓ | ✓ |
| AMNH6148 | F | 1894 | ✓ | ✓ |
| AMNH6149 | F | 1894 | ✓ |  |
| AMNH7973 | M | 1894 | ✓ | ✓ |
| YPM16268 | M | 2014 | ✓ | ✓ |
| YPM16876 | F | 2014 |  | ✓ |
| YPM16877 | F | 2014 |  | ✓ |
| YPM16924 | M | 2014 | ✓ | ✓ |
| YPM16267 | M | 2015 | ✓ |  |
| YPM16269 | F | 2015 | ✓ | ✓ |
| YPM16270 | F | 2015 | ✓ | ✓ |
| YPM16271 | M | 2015 | ✓ | ✓ |
| YPM16878 | F | 2015 | ✓ | ✓ |
| YPM16923 | F | 2015 | ✓ | ✓ |
| YPM16926 | M | 2015 |  | ✓ |
| YPM16927 | M | 2015 | ✓ | ✓ |
| YPM16930 | M | 2015 | ✓ |  |
| YPM16960 | F | 2015 | ✓ | ✓ |
| YPM16961 | M | 2015 | ✓ | ✓ |
| YPM16962 | F | 2015 |  | ✓ |
| YPM16964 | M | 2015 | ✓ | ✓ |
| YPM16965 | F | 2015 | ✓ | ✓ |
| YPM16967 | F | 2015 | ✓ | ✓ |
| YPM16968 | M | 2015 | ✓ | ✓ |
| YPM16969 | F | 2015 |  | ✓ |
| YPM16970 | M | 2015 | ✓ | ✓ |
| YPM16971 | F | 2015 | ✓ | ✓ |
| YPM16972 | F | 2015 |  | ✓ |
| YPM16973 | M | 2015 | ✓ | ✓ |
| YPM16974 | F | 2015 | ✓ | ✓ |
| YPM16975 | M | 2015 |  | ✓ |
| YPM16976 | M | 2015 | ✓ | ✓ |
| YPM16977 | F | 2015 |  | ✓ |
| YPM16874 | M | 2016 | ✓ | ✓ |
| YPM16883 | F | 2016 |  | ✓ |
| YPM16884 | M | 2016 | ✓ | ✓ |

**Table S2**- Cranial landmark abbreviations. Interlandmark distances are calculated as the Euclidean distance between two landmarks. See Figure S1 for landmark locations.

|  |  |  |  |
| --- | --- | --- | --- |
| **Orientation** | **Abbreviation** | **Landmark Name** | **Interlandmark Distances** |
| Both | OPI | Opisthion | LD |
| Both | RZ | Rostralmost end of the zygomatic plate- Right |  |
| Both | RZ | Rostralmost end of the zygomatic plate- Left |  |
| Both | SQ | Curve of Squamosal- Right |  |
| Both | SQ | Curve of Squamosal- Left |  |
| Dorsal | NSL | Nasale | IS, NA, PNS |
| Dorsal | NA | Nasion | NSL, BR |
| Dorsal | BR | Bregma | NA, SPI |
| Dorsal | SPI | Intersection between two parietals and interparietal bone | BA, LD |
| Dorsal | LD | Lambda | OPI, SPI |
| Dorsal | ZYGO | Anterior side of zygomatic arch-temoral suture- Left | EAM |
| Dorsal | IOS | Intersection of frontal suture with orbital rim- Right |  |
| Dorsal | LNS | Anterior most point along lateral zygomatic-frontal suture- Right |  |
| Dorsal | AMN | Anterior nasal-premaxilla junction- Right |  |
| Dorsal | ZYGO | Anterior side of zygomatic arch-temoral suture- Right |  |
| Dorsal | IOS | Intersection of frontal suture with orbital rim- Left |  |
| Dorsal | LNS | Anterior most point along lateral zygomatic-frontal suture- Left |  |
| Dorsal | AMN | Anterior nasal-premaxilla junction- Left |  |
| Ventral | IS | Intradentale Superior | NSL, PNS |
| Ventral | AIF | Anterior Incisive Foramen |  |
| Ventral | PIF | Posterior Incisive Foramen |  |
| Ventral | PNS | Posterior Nasal Spine | NSL, IS |
| Ventral | BA | Basion | EAM, APET |
| Ventral | LOC-L | Lateral Point on Ventral Margin of the Occipital Condyle- Left | LOC-R |
| Ventral | OA | Occipital auditory junction- Left |  |
| Ventral | APET | Anterior petrous temporal- Left | BA, PNS |
| Ventral | AIA | Anterior Inferior Auditory Bulla- Left |  |
| Ventral | AFO | Anterior Foramen Ovale- Left |  |
| Ventral | EAM | Anterior External Auditory Meatus- Left | BA, ZYGO |
| Ventral | PZA | Greatest curvature posterior edge of zygomatic process of temporal bone- Left |  |
| Ventral | PSA | Posterior superior alveoli- Left | ASA |
| Ventral | ASA | Anterior superior alveoli- Left | PSA |
| Ventral | PM | Maximal curvature of the malar process- Left |  |
| Ventral | LOC-R | Lateral Point on Ventral Margin of the Occipital Condyle- Right | LOC-L |
| Ventral | OA | Occipitalauditory junction- Right |  |
| Ventral | APET | Occipital auditory junction- Right | BA, PNS |
| Ventral | AIA | Anterior Inferior Auditory Bulla- Right |  |
| Ventral | AFO | Anterior Foramen Ovale- Right |  |
| Ventral | EAM | Anterior External Auditory Meatus- Right |  |
| Ventral | PZA | Greatest curvature posterior edge of zygomatic process of temporal bone- Right | BA, ZYGO |
| Ventral | PSA | Posterior superior alveoli- Right | ASA |
| Ventral | ASA | Anterior superior alveoli- Right | PSA |
| Ventral | PM | Maximal curvature of the malar process- Right |  |

**Table S3-** Mandible landmark abbreviations. Interlandmark distances are calculated as the Euclidean distance between two landmarks. See Figure S2 for landmark locations.

|  |  |  |
| --- | --- | --- |
| **Abbreviation** | **Landmark Name** | **Interlandmark Distances** |
| DA | Dorsal incisor alveolus | VA, VC, MA |
| MCR | Maximum curve on dorsal side of incisor ramus |  |
| MA | Anterior limit of m1 | DA, VA, VC |
| RT | Intersection of ascending ramus and tooth row |  |
| COR | Coronoid process dorsal limit | IDM, MCA, MCP, ANG |
| PIC | Posterior and inferior point of coronoid process |  |
| MCA | Anterior limit of condyle | COR, MCP, ANG, IDM |
| MCP | Posterior limit of condyle | COR, MCA, ANG, IDM |
| PAR | Posterior point of ascending ramus |  |
| ANG | Angular process posterior limit | COR, MCA, MCP, IDM |
| PA | Posteroventral tip of processus angularis |  |
| IDM | Maximum dorsal inflection between angular and alveolar processes | COR, MCA, MCP, ANG |
| PR | Posterior margin on ventral side of incisor ramus |  |
| VC | Ventral limit of chin | DA, VA, MA |
| VA | Ventral incisor alveolus | DA, VC, MA |

**Table S4**- Homogeneity of slopes test for cranium and mandible Procrustes ANCOVA slope vector length (top) and angle between slopes (bottom) for each pairwise combination of time period (1890s and 2010s) and sex (female, F and male, M), where the upper triangle shows *P*-values and lower triangle shows effect sizes (Z).

|  |  |  |  |  |  |
| --- | --- | --- | --- | --- | --- |
|  |  | 2010s : F | 2010s : M | 1890s : F | 1890s : M |
| **Slope Vector Length** | | |  |  |  |
| ***Crania*** | |  |  |  |  |
|  | 2010s : F | - | 0.864 | 0.579 | 0.512 |
|  | 2010s : M | -1.018 | - | 0.389 | 0.397 |
|  | 1890s : F | -0.433 | 0.144 | - | 0.111 |
|  | 1890s : M | -0.260 | 0.042 | 1.274 | - |
| ***Mandible*** | |  |  |  |  |
|  | 2010s : F | - | 0.454 | 0.693 | 0.647 |
|  | 2010s : M | -0.063 | - | 0.702 | 0.786 |
|  | 1890s : F | -0.650 | -0.689 | - | 0.939 |
|  | 1890s : M | -0.563 | -0.871 | -1.186 | - |
|  |  |  |  |  |  |
| **Slope Angle** | |  |  |  |  |
| ***Crania*** | |  |  |  |  |
|  | 2010s : F | - | 0.716 | 0.772 | 0.586 |
|  | 2010s : M | -0.682 | - | 0.768 | 0.601 |
|  | 1890s : F | -0.754 | -0.767 | - | 0.745 |
|  | 1890s : M | -0.363 | -0.361 | -0.727 | - |
| ***Mandible*** | |  |  |  |  |
|  | 2010s : F | - | 0.369 | 0.311 | 0.368 |
|  | 2010s : M | 0.174 | - | 0.662 | 0.340 |
|  | 1890s : F | 0.363 | -0.498 | - | 0.585 |
|  | 1890s : M | 0.165 | 0.256 | -0.345 | - |

**INPUT FILES**

Input files for fastsimcoal2 to estimate population size in Manhattan, NY brown rats.

**NYC.est**

// Priors and rules file

// *********************

[PARAMETERS]

//#isInt? #name #dist.#min #max

//all Ns are in number of haploid individuals

1 Nnyc unif 1 10000 output

[RULES]

[COMPLEX PARAMETERS]

**NYC.tpl**

//Parameters for the coalescence simulation program: fastsimcoal.exe

1

//Population effective sizes (number of genes=2*NumIndividuals)

Nnyc

//Samples sizes and samples age

248

//Growth rates : negative growth implies population expansion

0

//Number of migration matrices: 0 implies no migration between demes

0

//historical event: time, source, sink, migrants, new deme size, new growth rate, migration matrix index

0 historical event

//Number of independent loci [chromosome]

1 0

//Per chromosome: Number of contiguous linkage Block: a block is a set of contiguous loci

1

//per Block:data type, number of loci, per generation recombination and mutation rates and optional parameters

FREQ 1 0 2.5e-8 OUTEXP
